# Rheumatoid arthritis in remission: Decreased myostatin and increased serum levels of periostin

**DOI:** 10.1007/s00508-018-1386-0

**Published:** 2018-08-31

**Authors:** Katharina Kerschan-Schindl, Gerold Ebenbichler, Ursula Föeger-Samwald, Harald Leiss, Christina Gesslbauer, Malvina Herceg, Georg Stummvoll, Rodrig Marculescu, Richard Crevenna, Peter Pietschmann

**Affiliations:** 1 Department of Physical Medicine, Rehabilitation and Occupational Medicine, Medical University of Vienna, Waehringer Guertel 18–20, 1090 Vienna, Austria; 2 Department of Pathophysiology and Allergy Research, Center of Pathophysiology, Infectiology and Immunology, Medical University of Vienna, Vienna, Austria; 3 Department of Internal Medicine, Rheumatology, Medical University of Vienna, Vienna, Austria; 4 Department of Laboratory Medicine, Medical University of Vienna, Vienna, Austria

**Keywords:** Rheumatoid arthritis, Musculoskeletal markers, Physical function, Quality of life, Rheumatoide Arthritis, Muskuloskelttale Marker, Körperliche Funktion, Lebensqualität

## Abstract

**Background:**

Chronic inflammation of rheumatoid arthritis (RA) is associated with disturbances in muscle and bone metabolism.

**Aim:**

The purpose of this study was to investigate whether endocrine regulators of myogenesis and bone metabolism in patients with rheumatoid arthritis (RA) in remission differed from unaffected healthy controls. An additional point was whether these were associated with patients’ health-related functioning or particular bodily functions of the International Classification of Functioning, Disability and Health (ICF).

**Methods:**

Bone turnover and the markers for muscle, i.e. myostatin (MSTN), follistatin (FSTN), growth differentiation factor (GDF-15) and for bone, i.e. sclerostin (SOST), dickkopf 1 (Dkk1), periostin (PSTN) metabolism were determined in 24 female RA patients and matched healthy controls. The chair rising test (CRT), timed up and go test (TUG), 6 min walking test, maximum hand grip and back extensor strength tests were used to assess patients’ health-related functions. Additionally, bone mineral density of the lumbar spine and the hip region was measured.

**Results:**

For the bone turnover markers no differences were observed between patients and controls. In contrast, the markers MSTN and Dkk1 were significantly lower and FSTN and PSTN significantly higher in patients than controls. Patients performed worse in the CRT and TUG. Some correlations reflected associations between these endocrine factors and physical function.

**Conclusion:**

Anti-inflammatory therapy may be responsible for the positive effect on endocrine factors influencing myogenesis. Elevation of PSTN probably reflects the increased risk of fragility fractures in RA patients.

## Introduction

Rheumatoid arthritis (RA), a chronic inflammatory autoimmune disease primarily affecting the joints, is often accompanied by rheumatoid cachexia [1]. Fatigue, pain, restricted joint mobility, impaired muscle strength and decreased aerobic fitness limit patients’ physical functioning and reduce the quality of life. Biochemical parameters, performance-based tests and self-reported questionnaires may enable early detection of such limitations therefore enabling the early initiation of countermeasures. Additionally, in RA periarticular bone loss, bone erosion and systemic osteoporosis leading to an increased risk of fractures are often observed. In experimental as well as human studies it has been shown that the endocrine factors myostatin (MSTN; [2]) and its counterpart follistatin (FSTN; [3]) influence skeletal muscle growth in a negative [4, 5] and a positive way [6], respectively. The growth differentiation factor 15 (GDF-15), a transforming growth factor (TFG) beta family cytokine, has also been identified as a mediator of muscle wasting [7]. Sclerostin (SOST) and dickkopf-1 (Dkk 1) are inhibitors of the Wnt/beta-catenin signalling pathway and, thus, reduce bone formation and regeneration [8]. Periostin (PSTN), the osteoblast-specific factor 2 is another regulator of bone metabolism [9]. Changes of biochemical parameters, such as proinflammatory cytokines are well-known in inflammatory disease patients. Inflammation has a negative effect on muscle and bone metabolism. Nevertheless, serum levels of endocrine factors influencing myogenesis and bone homoeostasis have hardly been studied in RA patients so far.

A high disease activity is assumed to go hand in hand with a more pronounced deterioration of muscle and bone metabolism. Remission of RA is not very frequent. They ranged from 2% to 17% (Boolean definition; [10]). It was not the aim to investigate factors regulating myogenesis and bone metabolism in the majority of RA patients with high disease activity but to investigate these endocrine factors and possible associations with physical function in RA patients with low disease activity, e.g. postmenopausal RA patients in remission.

## Methods

### Study population

Female patients who presented for a routine health examination at the Rheumatology Department of Internal Medicine were invited to participate in the study. To be eligible, participants had to have RA according to the American College of Rheumatology (ACR) classification criteria from 1987 and had to be in remission according to the ACR/EULAR definitions of remission in RA clinical trials [11]. They further had to be postmenopausal, on a pharmacotherapy that had not been changed within the last 3 months and were either free or almost free from pain (ratings of ≤3 on an 11 points visual analogue scale). Patients were excluded if they had to undergo surgery within the past 3 months, had cancer, were immobilized, or had renal or liver insufficiency. For the control group, volunteers had to be healthy women who matched in age with a patient (±2 years) and did not regularly participate in strenuous physical activities. Ethical approval was provided by the Medical University of Vienna Ethics Committee and all participants gave written informed consent to this cross-sectional study after the procedure of the study was explained to them.

### Procedures

Venous blood samples were taken from patients with RA in remission as well as age-matched controls. Sample collection was performed in the morning to eliminate diurnal variations in biochemical variables. After centrifugation of the whole blood the serum was immediately frozen and stored at −70 °C until assayed. All samples were handled in a single batch run. Prior to the study examinations, anthropometric measures were performed. Standing height was measured in stockinged feet to the nearest centimeter using a stadiometer, and weight was measured using a balance beam scale, recalibrated monthly. Study participants conducted four different functional tests, filled in two questionnaires, and the bone mineral density was measured.

### Biochemistry

Biochemical analyses included the evaluation of serum calcium, phosphate, creatinine, gamma glutamyl transferase, parathyroid hormone, 25-OH-vitamin D, and alkaline phosphatase. Bone turnover markers were also studied: bone specific alkaline phosphatase (BAP; Liaison Analyzer, DiaSorin, Stillwater, MN, USA, detection limit: 0.1 µg/l, intra-assay coefficient of variation: 3.3–4.3%, inter-assay coefficient of variation: 6.1–8.1%), osteocalcin (Oc; Cobas 8000 Analyzer, Roche Diagnostics, Rotkreuz, Switzerland, detection limit: 0.01 ng/ml, intra-assay coefficient of variation: 0.9–1.3%, inter-assay coefficient of variation: 1.2–2.3%), N‑terminal propeptide of type I collagen (P1NP; Cobas 8000 Roche Analyzer, Roche Diagnostics, detection limit: 5 ng/ml, intra-assay coefficient of variation: 1.6–3.5%, inter-assay coefficient of variation: 2.0–3.8%), and cross-linked-C-telopeptide of type I collagen (CTX; Cobas 8000 Roche Analyzer, Roche Diagnostics, detection limit: 0.5 ng/ml, intra-assay coefficient of variation: 1.2–4.7%, inter-assay coefficient of variation: 1.5–5.7%). All analyses were conducted according to standard procedures. Additionally, the following musculoskeletal markers were investigated: myostatin (MSTN, colorimetric competitive immunoassay, Immundiagnostik, Bensheim, Germany, limit of blank LoB: 0.370 ng/ml, intra-assay coefficient of variation: <12%, inter-assay coefficient of variation: <14%, according to manufacturer’s data), follistatin (FSTN, colorimetric sandwich immunoassay, R&D Systems, Minneapolis, MN, USA, MDD (minimum detectable dose) range 0.005–0.068 ng/ml; mean MDD 0.016 ng/ml, intra-assay coefficient of variation: <3%, inter-assay coefficient of variation: <10%, according to manufacturer’s data), growth differentiation factor 1*5* (GDF-15, enzyme-linked immunosorbent assay, PromoCell, Heidelberg, Germany, MDD 2 pg/ml, intra-assay coefficient of variation: <10%, inter-assay coefficient of variation: <12%, according to manufacturer’s data), sclerostin (SOST, BI-20492, colorimetric sandwich immunoassays, Biomedica, Vienna, Austria; detection limit: 3.2 pmol/l, intra-assay coefficient of variation: ≤7%, inter-assay coefficient of variation: ≤10%, according to manufacturer’s data), dickkopf 1 (Dkk 1; BI-20412, colorimetric sandwich immunoassays, Biomedica; detection limit: 0.38 pmol/l; intra-assay coefficient of variation: ≤8.0%, inter-assay coefficient of variation: ≤12.0%, according to manufacturer’s data) and periostin (PSTN; SEH339Hu, colorimetric sandwich immunoassays, Cloud-Clone-Corp, Houston, TX, USA; detection limit: 0.068 ng/ml, intra-assay coefficient of variation: ≤10%, inter-assay coefficient of variation: ≤12%, according to manufacturer’s data).

### Functional assessment

Physical examination included different functional tests which were conducted by one of two trained assessors. Lower limb strength and mobility performance measurements comprised the chair rising test (CRT; [12]) and the timed up and go test (TUG; [13]). Completion of both tasks was timed from the beginning of the maneuver until the patient was re-seated. To assess functional exercise capacity the distance covered within 6 min of walking at maximum speed on a level course was measured (6 min walking test; 6MWT [14]). Both hands’ maximum isometric grip strength was obtained in a sitting position, with patients’ shoulders adducted to the body, their elbows flexed to 90 °, and their forearms and wrists in a neutral position. Subjects were instructed to press the Jamar dynamometer (Asimow Engineering, Los Angeles, CA, USA) as hard as possible three times on each side [15]. The mean of the best two values was noted. Back extensor muscle strength measurement included compilation of muscular endurance (Biering-Sörensen-Test; [16]) as well as maximum isometric trunk extension using the David® dynamometer (David Helath Solution Ltd, Helsinki, Finnland).

Quality of life was assessed with a disease-specific and a generic questionnaire, the Health Assessment Questionnaire [17] and the MOS SF-36 [18, 19].

### Bone mineral density measurement

The dual energy X‑ray absorption method (DXA; HOLOGIC 4500; Hologic, Waltham, MA, USA; coefficient of variation (CV): 2%) was used to measure bone mineral density (BMD) of the lumbar spine and the femoral neck.

## Statistical analysis

Data are presented as medians and quartiles. Statistical analysis of between group differences concerning the serum levels of the biochemical markers as well as the parameters of physical function was performed with the Mann-Whitney U-test. Spearman’s rank correlation coefficient was used to identify potential associations between biochemical markers and physical function. Significance was set at *p*-values less than 0.05. Statistical analyses were done using the software packages GraphPad Prism 5 (Prism 5 for Windows, Version 5.00, 2007) and SPSS Statistics V21 (SPSS, Chicago, IL, USA, 2012).

## Results

A total of 24 female patients suffering from RA in remission and 24 age and gender matched healthy controls with a median age of 61 years [58; 70 years] and 62 years [58; 68 years], respectively were included in this cross-sectional study. There were no significant differences between the RA and control group with respect to age, height (162 [157.0; 166.0] cm vs. 160 [158.5; 165.3] cm), and weight (69 [64; 82] kg vs. 63 [61; 75] kg). Symptom duration in the RA group was 62 [45; 99] months. Most patients (*n* = 21) received disease-modifying antirheumatic drugs (DMARDs), 2 patients did not need any disease-specific medication and 1 patient each took chloroquine phosphate and sulfasalazine, respectively. The patients’ median Clinical Disease Activity Index (CDAI; [20]) was 1.0 [0.4; 1.6] and the median Simplified Disease Activity Index (SDAI; [20]) 1.5 [0.9; 2.3]. Of the patients 80% were seropositive with a median rheumatoid factor of 47.6 [22.5; 120.5].

### Blood laboratory tests

All findings from the biochemical blood tests are provided for both patients and controls in Table 1. Serum levels of phosphate were significantly lower in RA patients than in controls.

**Table 1:** Biochemical parameters of patients and controls

	RA patients (*n* = 24)	Controls (*n* = 24)	*p*
Ca (mmol/l)	2.37 [2.30; 2.45]	2.44 [2.39; 2.50]	n.s.
Ph (mmol/l)	1.04 [0.97; 1.16]	1.21 [1.13; 1.27]	<0.001
Creatinine (mg/dl)	0.82 [0.63; 0.94]	0.88 [0.74; 0.93]	n.s.
GGT (U/l)	20.5 [14.3; 34.0]	18.5 [15.3; 25.8]	n.s.
PTH (pg/ml)	44.4 [35.1; 57.5]	40.4 [31.8; 53.1]	n.s.
25-OH-vitamin D (mmol/l)	74 [54; 82]	69 [57; 87]	n.s.
AP (U/l)	81.0 [73.0; 88.0]	69.0 [57.5; 86.0]	n.s.
BAP (ng/ml)	13.7 [11.3; 15.3]	12.9 [9.6; 17.5]	n.s.
Oc (ng/ml)	22.4 [16.0; 25.3]	23.9 [20.7; 30.1]	n.s.
P1NP (ng/ml)	41.5 [36.3; 52.5]	51.5 [40.0; 58.5]	n.s.
CTX (ng/ml)	0.27 [0.21; 0.34]	0.32 [0.27; 0.39]	n.s.
Myostatin (ng/ml)	37.4 [29.9; 44.0]	49.7 [45.3; 57.3]	<0.001
Follistatin (pg/ml)	2054 [1696; 2655]	1588 [1259; 1811]	0.004
MSTN/FSTN	0.02 [0.01; 0.02]	0.03 [0.02; 0.04]	<0.001
GDF-15 (pg/ml)	643.0 [534.7; 1076.2]	586.3 [430.1; 666.1]	n.s.
Sclerostin (pg/ml)	28.2 [25.2; 33.1]	29.0 [23.4; 41.1]	n.s.
Dickkopf 1 (pg/ml)	27.9 [20.4; 44.6]	45.8 [32.0; 58.7]	0.004
Periostin (ng/ml)	3063 [2359; 4203]	1021 [807; 1941]	<0.001

Data are given as median [quartiles]

*Ca* calcium, *Ph* phosphate, *GGT* gamma-glutamyl transferase, *PTH* parathyroid hormone, *25-OH-vitamin D* 25-hydroxy-vitamin D, *AP* alkaline phosphatase, *BAP* bone-specific alkaline phosphatase, *OC* osteocalcin, *P1NP* propeptides of type I collagen, *CTX* telopeptides of collagen type I, *MSTN/FSTN* myostatin/follistatin, *GDF-15* growth differentiation factor 15, *n.s.* not significant

For the bone turnover markers, no group-specific differences could be detected; however, serum levels of the muscle regulators MSTN and FSTN differed significantly between groups. The mean MSTN level was lower and FSTN higher in RA patients than in their age matched healthy controls, thus leading to a reduced MSTN/FSTN ratio in RA patients. The serum levels of GDF-15, however, did not differ between the two groups. The mean Dkk 1 level was significantly lower in RA patients. No such group-specific difference was identified for SOST but PSTN was significantly higher in RA patients.

### Functional tests

As shown in Table 2, four out of the seven functional tests significantly differed between patients and healthy controls. As such RA patients performed significantly worse in the CRT evaluating lower extremity muscle power and the TUG, a test for balance, as well as the 6MWT and the Biering-Sörensen test evaluating muscular endurance of the back extensors. Concerning grip strength and isometric muscular strength of the back extensors no group-specific differences could be detected.

**Table 2:** Assessment of differences in functional status and quality of life between patients and controls

	RA patients (*n* = 24)	Controls (*n* = 24)	*p*
CHT	11.2 [9.0; 14.8]	7.1 [6.1; 7.9]	<0.001
TUG	7.6 [7.0; 8.7]	4.9 [4.4; 5.7]	<0.001
6MWT	521 [447; 590]	650 [605; 683]	<0.001
Grip strength left	15.5 [10.3; 21.8]	18.5 [14.8; 21.3]	n.s.
Grip strength right	18.0 [12.5; 22.3]	19.0 [16.3; 24.3]	n.s.
Back extensors: endurance	43 [15.7; 62]	118 [86; 184]	<0.001
Back extensors: strength	159 [126; 186]	157 [120; 193]	n.s.
HAQ	0 [0; 4]	0 [0; 0]	0.001
MOS-SF 36			
Physical functioning	90 [80; 98]	100 [95; 100]	0.008
Role, physical	100 [100; 100]	100 [100; 100]	n.s.
Role, emotional	100 [100; 100]	100 [100; 100]	n.s.
Social functioning	100 [88; 100]	100 [88; 100]	n.s.
Mental health	84 [72; 88]	80 [68; 90]	n.s.
Bodily pain	80 [62; 100]	100 [74; 100]	0.05
Vitality	70 [50; 75]	75 [60; 80]	n.s.
General health	72 [58; 77]	80 [67; 92]	0.042

Data are given as median [quartiles]

*CHT* chair rising test, *TUG* timed up and go test, *6MWT* 6 min walking test, *HAQ* Health Assessment Questionnaire, *MOS SF-36* Medical Outcomes Study Short Form-36, *n.s.* not significant

The HAQ scores were significantly lower in RA patients compared to healthy controls. In 3 of the 8 domains, the MOS SF-36 revealed a reduced quality of life in RA patients: physical functioning, mental health, and general health (Table 2).

The BMD measurement of the femoral neck was found to be slightly but not significantly lower in RA patients than in healthy controls with median T scores of −1.2 [−1.9; −1.2] in RA patients and −0.6 [−1.4; −0.1] in controls. For the lumbar spine, BMD scores were found to be normal both in RA patients and controls with the median T scores of −1.2 [−2.3; 0.5] and −1.1 [−2.2; −0.5], respectively.

The relationship of the different musculoskeletal markers to each other and the results of explorative correlation analyses between these markers and physical function are shown in Table 3. High serum levels of MSTN were associated with low grip strength and a high HAQ score. Serum levels of FSTN were positively correlated with the CRT, the TUG, and the HAQ but negatively with the 6MWT and the Biering-Sörensen test. A positive association was detected between GDF-15 levels and the CRT, the TUG, as well as the HAQ; the association with the 6MWT and the Biering-Sörensen test was negative. Serum levels of the two negative regulators of the Wnt signalling pathway did not show any association with physical function. The correlation between PSTN and the CRT, the TUG as well as the HAQ was positive whereas it was negative with the 6MWT, the Biering-Sörensen test, and right hand grip strength.

**Table 3:** Correlation analyses of musculoskeletal markers and physical function

	MSTN	FSTN	MST/FSTN	GDF-15	Dkk1	SOST	PSTN
MSTN	1	0.076	0.698**	0.086	−0.60	0.042	0.260
FSTN	0.076	1	−0.559**	0.202	0.51	−0.33	0.337*
MSTN/FSTN	0.698**	−0.559**	1	−0.063	−0.103	0.068	−0.100
GDF-15	0.086	0.202	−0.063	1	−0.126	−0.027	0.345*
PSTN	0.260	0.337*	−0.100	0.345*	−0.321*	−0.296*	1
Dkk1	−0.60	0.51	−0.103	−0.126	1	0.205	−0.321*
SOST	0.042	−0.333	0.068	−0.027	0.205	1	−0.296*
PSTN	0.260	0.337*	−0.100	0.345*	−0.321*	−0.296*	1
Chair rise	0.221	0.328*	−0.073	0.473**	−0.282	0.058	0.606**
TUG	0.098	0.561**	−0.302*	0.464**	−0.186	−0.052	0.536**
6MWT	−0.030	−0.394**	0.225	−0.397**	0.219	0.142	−0.476**
Biering S	−0.059	−0.384**	0.216	−0.329*	0.211	0.190	−0.332*
Grip strength left	−0.370**	−0.225	−0.117	−0.003	−0.16	0.093	−0.240
Grip strength right	−0.318*	−0.192	−0.117	−0.06	0.080	0.115	−0.359*
HAQ	0.492**	0.452**	0.069	0.487**	−0.225	−0.117	0.562**

*MSTN* myostatin, *FSTN* follistatin, *MSTN/FSTN* Myostatin/Follistatin, *GDF-15* growth differentiation factor 15, *Dkk1* dickkopf 1, *SOST* sclerostin, *PSTN* periostin, *TUG* timed up and go test, *6MWT* 6min walking test, *Biering S* Biering-Sörensen test, *HAQ* Health Assessment Questionnaire

*p < 0.05, **p < 0.01

## Discussion

In RA patients, formation and breakdown of muscle and bone are assumed to be unbalanced. Assessment of endocrine factors influencing myogenesis and bone homoeostasis are important for the development of counteracting strategies. For the first time this study evaluated such endocrine factors and their relationship with physical function in RA patients in remission. Findings revealed 1) that the MSTN/FSTN ratio and serum levels of Dkk1 were significantly lower whereas PSTN values were elevated in RA patients when compared with controls, 2) physical functioning of RA patients was found to be impaired in some functional tests and 3) there were significant correlations between the endocrine factors MSTN and FSTN and physical function tests.

Compared with age-matched controls, the serum levels of the negative regulator of muscle mass, MSTN were found to be significantly lower in RA patients. These findings seem to contrast observations from chronic inflammatory diseases which are associated with a loss of muscle mass, rheumatoid cachexia [1] or from an experimental study that has demonstrated unaffected MSTN expression by arthritis [21]. No human investigation exists so far. Consistent with the lower than normal MSTN level in RA patients, FSTN, the antagonist of MSTN was significantly elevated in the RA patients studied when compared to controls. Indeed, the follistatin-related protein gene (FRP/FSTL1) mRNA has been demonstrated to be overexpressed in the synovium of RA patients [22]. The MSTN/FSTN ratio was significantly reduced in RA patients compared with controls. Since almost all patients were on biologicals, it could well be that these immune modulating therapies have a positive effect on the regulators of muscle mass, thereby decreasing MSTN and increasing FSTN. Pro-inflammatory cytokines, such as tumor necrosis factor (TNF) alpha are supposed to be involved in the pathogenesis of rheumatoid cachexia. These cytokines activate nuclear factor κB and thus lead to an increased proteolysis of muscle cells via the ubiquitin-proteasome pathway [23]. With DMARDs there is the possibility to effectively suppress disease activity. This reduction of inflammation seems to positively influence muscle mass and function in RA patients, an effect which has previously been shown in “inflammaging”-associated sarcopenia [24].

Serum levels of the negative regulator of muscle mass GDF-15 were similar in both groups. This finding is in contrast to the study by Tanrikulu et al. [25] who recently found elevated GDF-15 levels in 46 RA patients compared with controls. After splitting the group into the patients with active and nonactive RA, however, they showed that serum levels of GDF-15 were significantly higher in patients with active disease. The prsent study population included only RA patients in remission.

The two inhibitors of the Wnt signalling pathway show diverse results. No group-specific difference was identified for SOST but serum levels of Dkk 1 were significantly lower in RA patients compared with controls, probably indicating a repression of bone formation and regeneration in RA patients in remission. This effect may also be a positive epiphenomenon of the therapy with biologicals because reduction of inflammation influences bone metabolism in a positive way, thereby likely facilitating the markers of the Wnt signalling pathway towards values of healthy controls. Adami et al. [26] have shown that the initiation of anti-TNFapha treatment leads to a decrease in Dkk 1 serum levels in RA patients. The SOST serum levels did not change in their study group. Thus, our data are similar to the results of Adami et al. The PSTN level was significantly higher in the group of RA patients. This result is in line with the OFELY study [27], which showed that postmenopausal women (without rheumatoid arthritis) have increased serum levels of PSTN and an associated risk of fragility fractures. In lung transplantation recipients increased levels of PSTN have also shown (manuscript in preparation). Lung transplantation recipients as well as RA patients have an increased risk of fracture. This is the first study evaluating serum levels of PSTN in patients suffering from RA. The very low HAQ score of RA patients underlines the marginal limitations these patients have in their activities of daily living. Nevertheless, compared to controls, significant differences exist in some functional tests and in three domains of the MOS SF-36. Thus, physical function is partially limited in RA patients in remission.

According to the correlation analyses, serum levels of MSTN were only significantly associated with grip strength. The fact that MSTN is a regulator of muscle mass and not muscle function may be responsible for the lack of association with other functional tests. Additionally, experimental data showed that because of the loss of oxidative characteristics the force production of the skeletal muscle of a MSTN knockout mouse is compromised [28] and the aerobic performance is impaired [29]. The 6MWT and the Biering-Sörensen test evaluate muscular endurance. As FSTN is secreted by several cell types [30] it is not as muscle-specific as MSTN. That could probably explain the inconsistent correlations between the serum levels of FSTN and the functional tests. The positive correlations between GDF-15 and the CRT as well as the TUG test and the negative correlations between this mediator of muscle wasting on the one hand and the 6MWT as well as the Biering-Sörensen test on the other hand reflect associations in the expected way. The correlations between PSTN and physical function confirm the link between muscle and bone. Unexpectedly, RA patients had significantly lower serum levels of phosphate compared with controls; however, all values were within the normal range and there was no evidence of hyperparathyroidism, vitamin D deficiency, or renal dysfunction.

The limitations of this study are the cross-sectional design and the relatively small number of patients investigated but the design was chosen because of the explorative character of this pilot study. Of course, assessing electromyography parameters during muscle contraction would have been interesting but unfortunately, this was not possible. The study’s strength is the careful selection of patients with RA in remission.

In conclusion, RA patients in remission have reduced serum levels of MSTN and elevated levels of FSTN compared with healthy controls. The inhibitor of the Wnt signalling pathway Dkk1 is reduced as well. The anti-inflammatory effect of the DMARD therapy may be responsible for these findings. Despite the unexpected positive findings of the endocrine factors influencing myogenesis, patients with RA in remission were still partially limited in physical function. This first evaluation of serum levels of PSTN in RA patients showed significantly elevated values, which probably reflect the increased risk of fragility fractures in these patients. Further research is needed to identify the most relevant factor in concerns of association with physical function in a larger collective of RA patients in remission.
